# Can volunteering in later life reduce the risk of dementia? A 5-year longitudinal study among volunteering and non-volunteering retired seniors

**DOI:** 10.1371/journal.pone.0173885

**Published:** 2017-03-16

**Authors:** Yannick Griep, Linda Magnusson Hanson, Tim Vantilborgh, Laurens Janssens, Samantha K. Jones, Martin Hyde

**Affiliations:** 1 Department of Psychology, University of Calgary, Calgary, Canada; 2 Stress Research Institute, Stockholm University, Stockholm, Sweden; 3 Work and Organizational Psychology (WOPs), Vrije Universiteit Brussel, Brussel, Belgium; 4 Faculty of Medicine, KU Leuven, Leuven, Belgium; 5 Centre for Innovative Ageing, Swansea University, Swansea, United Kingdom; Istituto Di Ricerche Farmacologiche Mario Negri, ITALY

## Abstract

We propose that voluntary work, characterized by social, physical and cognitive activity in later life is associated with fewer cognitive problems and lower dementia rates. We test these assumptions using 3-wave, self-reported, and registry data from the 2010, 2012, and 2014 Swedish National Prescribed Drug Register. We had three groups of seniors in our data: 1) no volunteering (*N* = 531), 2) discontinuous volunteering (*N* = 220), and 3) continuous volunteering (*N* = 250). We conducted a path analysis in Mplus to investigate the effect of voluntary work (discontinuously and continuously) on self-reported cognitive complaints and the likelihood of being prescribed an anti-dementia treatment after controlling for baseline and relevant background variables. Our results indicated that seniors, who continuously volunteered, reported a decrease in their cognitive complaints over time, whereas no such associations were found for the other groups. In addition, they were 2.44 (95%CI [1.86; 3.21]) and 2.46 (95%CI [1,89; 3.24]) times less likely to be prescribed an anti-dementia treatment in 2012 and 2014, respectively. Our results largely support the assumptions that voluntary work in later life is associated with lower self-reported cognitive complaints and a lower risk for dementia, relative to those who do not engage, or only engage episodically in voluntary work.

## Introduction

As people age, the risk of dementia increases substantially [1]. Delaying dementia onset by a few years would therefore have enormous benefits for the health and social care sectors, and would have huge implications for the psychological well-being of the patients and their loved-ones [2]. Following the work of Fratiglioni and colleagues [3], who demonstrated that social, physical, and cognitive activity protects retired seniors from cognitive decline, we introduce voluntary work—defined as 1) activities performed out of free will, 2) without receiving remuneration, 3) in a formal organization, and 4) benefiting others [4]—as a specific form of later-life activity that may protect retired seniors from cognitive complaints and dementia. The theoretical tenet behind this assumption is that volunteering provides retired seniors with a clear time structure, increases social, physical, and cognitive activity, and allows them to participate in a collective purpose (all to varying degrees depending on the nature of the voluntary work) [5]. The unique combination of these aspects and forms of activity may subsequently—through social, physical, and cognitive mechanisms—lead to improved cognitive functioning and reduced dementia risks [5].

Several scholars have found support for the notion that *social* [6,7], *physical* [8–11], and/or *cognitive* [12–14] activity in later life is associated with a reduced risk of dementia. Across these studies, risks of dementia were reduced by 26% to 60% over a 4- to 9.5-year follow up. Some scholars even suggested that the *interplay* between social, physical, and cognitive activity—such as characterized in voluntary work—is most beneficial in reducing memory impairment and dementia risk in later life [3, 15, 16]. Retired seniors who engaged in activities that demanded moderate effort in all, or at least two of the three domains were 47% less likely to develop dementia. Fratiglioni and colleagues [3] argued that a physically active, cognitively challenging, and socially integrated lifestyle in late life protects against dementia. Despite these astute and novel insights, cohesive evidence for the protective benefits associated with volunteering in later life is lacking.

Recently, Anderson and colleagues [17] proposed that “Given the emerging evidence that seniors engaged in formal volunteering develop fewer functional limitations and improved memory and executive functioning, it is a reasonable and important hypothesis that giving one’s time and skills to productive activity will also help reduce risk of dementia. The time to test this hypothesis is now” (p.24). Based on this recent call and the above presented arguments, this study examines the relationship between volunteering and (1) self-reported cognitive complaints, and (2) independent indicators of anti-dementia treatment from the Swedish National Prescribed Drug Register among retired seniors (65 years or older). Specifically, we followed and compared those who engaged in voluntary work continuously (i.e., those individuals who volunteer throughout the entire study), discontinuously (i.e., those individuals who volunteer sporadically throughout the entire study), and non-volunteering retired seniors over a 5-year period. In what follows we will provide an extensive review of the literature on social, physical, and cognitive mechanisms and cognitive health in later life before presenting the hypotheses.

### Volunteering in later life: A protective factor for dementia?

In general, a range of (prospective cohort) studies have reported a positive relationship between social, physical, and cognitive activity in later life and lower dementia prevalence, even after controlling for potentially confounding variables such as age, gender, years of education, the type of activity, depression, baseline cognitive functioning, the number of chronic diseases, early-life influences, genetics, family history of dementia, coronary heart disease, vascular risk factors, physical functioning, cerebrovascular disease, and apoliproprotein E genetype epsilon4 allele [3].

From a *social mechanism perspective*, engaging in voluntary work in later life substitutes for some of the latent functions of employment, such as social contact outside of the family and social status, which may have been lost due to retirement, widowhood or reduced parental roles [18, 19]. For example, based on the work of Bennett and colleagues [6] and Fratiglioni and colleagues [7], it could be argued that engaging in voluntary work provides one with a broader social network (i.e., more social contacts outside of the family), which holds the potential to mitigate the relationship between dementia and cognitive functioning; a poor or limited social network increased the risk of dementia by 60%.

When it comes to the *physical mechanism*, it has been theorized and empirically supported, that the protective effect of voluntary physical activity is the result of increased levels of a brain-derived neurotrophic factor, stimulated neurogenesis in the hippocampus, increased resistance to brain injury, improved learning and better brain plasticity [20]. Indeed, Colcombe and colleagues [8] and Erickson and colleagues [9] found that physical activity in later life resulted in significant increases in both gray and white matter regions and a 2% increase in the anterior hippocampus. These changes might result in a better cognitive functioning and a reduced risk of dementia. In addition, physical exercise for at least three times per week was associated with a 42.3% improvement in learning and memory over a 5-year follow-up period [10], and reduced the risk of dementia with 34% over 6.2 years [11]. Hence, it can be argued that the physical activity that accompanies the enactment of voluntary work (e.g., increased walking, interacting with other individuals, gardening, cooking and serving food, etc) [5, 19] reduces the risk of being diagnosed with dementia.

Finally, in terms of the *cognitive mechanisms*, engaging in activities that require cognitive capacities increased the use of cognitive skills and functional reorganization, and induced neurogenesis, synaptogenesis, and cortical plasticity [21–23]. By doing so, these cognitive activities allow one to maintain his/her level of cognitive functioning when aging [24]. Indeed, engaging in activities that stimulate cognitive functioning resulted in a 26% to 51% reduced risk of dementia over a 4-year follow up [12, 13].

Few studies, with the exception of two, have examined the *interplay between social*, *physical*, *and cognitive activity* in later life. Carlson and colleagues [15] found that engaging in a greater variety of leisure activities, regardless of the cognitive challenge of these activities, was associated with an 8 to 11% reduction in the risk of memory impairment and global cognitive outcomes 9.5 years later. Karp and colleagues [16] found that seniors who engaged in activities that required moderate social, physical, or cognitive efforts—compared to activities that required little or no effort—were respectively 32%, 39%, and 29% less likely to develop dementia over the course of 6 years. The most beneficial effect was found for seniors who engaged in activities that demanded moderate effort *in all or in at least two of the three domains*, being 47% less likely to develop dementia. Fratiglioni and colleagues [3] concluded that a physically active, cognitively challenging, and socially integrated lifestyle in late life protects against dementia. Given the proposed increased social, physical, and cognitive activity that accompanies the enactment of voluntary work [5, 17], voluntary work should protect retired seniors from developing cognitive complaints and dementia. Therefore, we hypothesize:

*Hypothesis 1*: Retired seniors who were continuously more actively engaged in voluntary work (more hours of voluntary work at time T1) are less likely to report cognitive complaints (time T2 and T3) compared to retired seniors who do not volunteer, or who volunteer discontinuously.

*Hypothesis 2*: Retired seniors who were continuously more actively engaged in voluntary work (more hours of voluntary work at time T1) are less likely to be prescribed an anti-dementia treatment (time T2 and T3) compared to retired seniors who do not volunteer, or who volunteer discontinuously.

## Methods

### Procedure

We conducted a 5-year follow-up study using survey data from the 2010 (time T1), 2012 (time T2), and 2014 (time T3) waves of the Swedish Longitudinal Occupational Survey of Health (SLOSH). SLOSH is a nationally representative longitudinal cohort survey of the Swedish population commissioned by the Stress Research Institute at Stockholm University and approved by the Regional Research Ethics Board in Stockholm and the Research Ethics Committee at Karolinska Institutet. At all points in time, we provided written information to all participants, informing them about the purpose of the study, the discretionary nature of participation, the confidential treatment of the data, and the possibility to withdraw from the study. We requested participants to complete and return—using a pre-stamped envelope—a questionnaire. The data were fully anonymized and could not be traced back to specific individuals, or limited groups of people.

### Ethics statement

The SLOSH project is carried out within the framework of Stockholm Stress Centre, a FAS centre of excellence (grant #2009–1758). SLOSH and register linkages are covered by existing approvals from the Regional Research Ethics Board in Stockholm (Dnr 2006/158-31; 2008/240-32 extended 2008-07-01; Dnr 2008/1808-32; Dnr 2009/337-32; Dnr 2009/493–31/3; and Dnr 2010/0145-32) and earlier from the research ethics committee at Karolinska Institutet (1992-09-21, dnr 92–198, extended 2000-11-15, same Dnr, plus 2003-03-10, Dnr 03–125). SLOSH is furthermore approved internally by Statistics Sweden (SCB # 24/9784/2001, # 115894/820137-8, and # 858758-6/198 633) and by the National Board of Health and Welfare (SoS). This specific study is covered by approval from the Medical Research Ethics Board in Brussels (B.U.N. 143201422457).

### Participants

In total, 7,222 individuals returned a filled-out questionnaire across all SLOSH waves (attrition rate between 2010 and 2012 = 3.33%; attrition rate between 2012 and 2014 = 9.89%). We focused on formal volunteering in relation to cognitive health among retired seniors and thus only retained respondents aged 65 or older who completed the surveys (*N* = 1,001). Our sample comprised three groups of retired seniors: (1) no volunteering (*N* = 531), (2) discontinuously volunteering (*N* = 220), and (3) continuous volunteering (*N* = 250). Discontinuous volunteers are those individuals who volunteered during one or two waves out of all the SLOSH waves, whereas continuous volunteers are those individuals who volunteered across all three waves. We conducted logistic regression analyses to estimate differences between our final sample and dropouts. None of the demographics nor the variables under study explained dropout.

At the 2010 SLOSH wave, 94.4% had Swedish nationality, 50.4% were female, their mean age was 67.75 years (*SD* = 1.68), 78.4% were married or cohabiting, 93.4% had no children living with them, their mean combined family income was 37,221 US dollar (*SD* = 20,532 US dollar), 33.5% had obtained a university degree, 45.2% had a higher educational degree, and 21.3% had a high school degree. Those who volunteered did this on average 5.88 hours (*SD* = 5.67) per week. During the 2012 and 2014 SLOSH wave we only had data on our respondents’ gender, age, income, and hours spend volunteering. In 2012, 49.3% were female, their mean age was 69.95 years (*SD* = 1.66), and their mean combined family income was 33,386 US dollar (*SD* = 19,395 US dollar). Those who volunteered did this on average 5.64 hours (*SD* = 5.48) per week. In 2014, 50.3% were female, their mean age was 71.93 years (*SD* = 1.66), and their mean combined family income was 32,471 US dollar (*SD* = 18,454 US dollar). Those who volunteered did this on average 5.83 hours (*SD* = 5.27) per week.

### Measures

#### Independent variables

*Voluntary work* was measured with the following question: “How many hours in an average week did you spend on voluntary (unpaid) work since the previous survey in, for example, a society, relief organization, religious organization, political party or non-profit organization?” The number of hours of voluntary work was used as an independent variable. Based on this variable, we also created a dummy variable representing the *different voluntary work groups* (i.e., continuous, discontinuous, and no volunteering) to check for differences in the hypothesized relationships across the voluntary work group.

#### Dependent variables

*Self-reported cognitive complaints* were measured with four items of The Copenhagen Psychosocial Questionnaire II (COPSOQ II), reflecting the core symptoms of cognitive complaints: ‘problems concentrating’, ‘difficulty making decisions’, ‘difficulty remembering’, and ‘difficulty thinking clearly’ [25]. Response options ranged from ‘never’ (0) to ‘always’ (4). Cronbach’s alpha coefficients were satisfactory (α_2010_ = .89; α_2012_ = .87; α_2014_ = .87).

*Data on anti-dementia treatments (i*.*e*., *medication)* were retrieved from the Swedish National Prescribed Drug Register; a patient-based register that contains data on dispensed out-patient prescriptions at all Swedish pharmacies, excluding drugs sold over-the-counter and inpatient use in hospitals [26]. Data on the type of anti-dementia treatment were matched to the survey respondents, using their anonymized personal identification number. We extracted all redeemed prescriptions used in the treatment of dementia as from 2010 until 2014 (biannual purchases from e.g. 1^st^ of January 2010 to 31^st^ of December 2011). These were: Donepezil, Rivastigmine, Galantamine, and Memantine. All of these anti-dementia medications (with the exception of memantine) are acetylcholinesterase inhibitors used to increase the level of acetylcholine, a chemical in the brain that is low in people with dementia. Memantine is an N-methyl-D-aspartate antagonist that improves neural functioning by reducing the amount of glutamate uptake in the post-synaptic neuron, which reduces the rate of intra-cellular calcium accumulation and, by doing so, slows down the damage to brain cells affected by dementia [27].

We focused on *the likelihood of being prescribed an anti-dementia treatment*. To capture this likelihood, we created a dummy variable with value “one” when respondents were prescribed an anti-dementia treatment, and value “zero” when respondents were not prescribed an anti-dementia treatment. Based on this, we could predict the likelihood that one would be prescribed an anti-dementia treatment. Eighteen (prevalence of 0.018), twenty-two (prevalence of 0.022), and twenty-two (prevalence of 0.022) respondents were prescribed an anti-dementia treatment in 2010, 2012, and 2014, respectively. These prevalence rates are slightly smaller than the prevalence rates reported by a revision of population-based cohort prevalence rates of dementia in the elderly in Europe [28]. Specifically, Lobo and colleagues [28] found that the prevalence of dementia increased continuously with age and was 0.8% in the group age 65 to 69 years (mean age in our sample was 67.75 years at onset of the study).

#### Control variables

Based on the proposed influence of several background variables on the development of cognitive problems and dementia rates [29–31] we included age, educational level, income, general self-rated health, prolonged sickness or disability, and levels of general activity as control variables. *Age* was measured in years. Respondents indicated the highest *education degree* they had attained. *Income* was assessed in thousands of Swedish Crowns. *General self-rated health* was measured by asking respondents to rate their general state of health on a five-point Likert scale ranging from ‘very poor’ (1) to ‘very good’ (5). We asked respondents if they had some *prolonged sickness*, *accident-related complaints*, *a disability or other weaknesses* (0 = no, 1 = yes) [9]. Finally, we asked respondents to indicate how much *exercise* they get in an average week. Response options ranged from ‘I never exercise’ (0) to ‘I exercise regularly’ (3).

### Analysis

We conducted a path analysis in Mplus version 7.1 [32] to test our hypotheses. Path analysis extends conventional regression analysis because it allows for the simultaneous, instead of single, estimation of path coefficients from the amount of hours spent volunteering each week to one’s self-reported cognitive complaints and prescribed anti-dementia medication (i.e., the likelihood and the strength of the dosage) in 2012 and 2014 [33]. Our data on the likelihood of being prescribed an anti-dementia treatments followed a zero-inflated Poisson distribution, in which most observations are zero (i.e., the absence of being prescribed an anti-dementia treatment) and few observations are one (i.e., the presence of being prescribed an anti-dementia treatment) [34]. Hence, we defined the likelihood of being prescribed an anti-dementia treatment as a zero-inflated Poisson variable during the analyses. By doing so, we 1) more accurately model the distribution of the variable as one in which the occurrence of an event (i.e., diagnosis of dementia) is a rare event, 2) prevent these events from being outliers that may pull the association between anti-dementia treatment (and cognitive complaints) and hours of volunteering as would be the case in traditional ways of analyzing the data (i.e., not accounting for the zero-inflated nature of the data), and 3) allow for the estimation of rare, yet very important, observations such as the likelihood of being diagnosed with dementia.

We used the option TYPE = MIXTURE to estimate a multi-group model for the continuous and discontinuous voluntary work groups. Note that we do not add the “no volunteering” group to the multi-group model because hours of voluntary work equals zero for all respondents. Additionally, we modeled *change* in the outcome variables. This is achieved by introducing the outcome variable measured at an earlier point in time as a control variable. By doing so, we controlled for baseline self-reported cognitive complaints and prescribed anti-dementia medication. As a consequence, changes in the outcome variables are not influenced by significant baseline differences or by previous measurements of the same variable at an earlier point in time. In this path model we allowed for (1) all outcome variables in 2012 and 2014 to be correlated, (2) the control variables in 2010 to be correlated with the outcome variables in 2012 and 2014, and (3) all outcome variables in 2012 and 2014 to be correlated with themselves at an earlier point in time.

However, it could still be argued that those who experienced more cognitive complaints or were prescribed an anti-dementia treatment might be less likely to start volunteering in the future. To rule out any concerns regarding reversed causation, we estimated a reversed causation path model. In this path model, we predicted one’s voluntary activity in 2012 and 2014 based on one’s cognitive complaints and the likelihood of being prescribed an anti-dementia treatment in 2010 and 2012. In addition, we allowed correlations between (1) one’s voluntary activities across all waves, (2) one’s cognitive complaints and the likelihood of being prescribed an anti-dementia treatment across all waves, and (3) one’s voluntary activities were allowed to correlate with themselves at an earlier point in time to model change.

## Results

### Descriptive statistics

Table 1 provides an overview of the means, standard deviations, and correlations of all variables under study across all individuals in the study. The reported means for previous illnesses or complaints and anti-dementia treatment likelihoods refer to the percentage of respondents who indicated that they were suffering from a previous illness or complaint and were prescribed an anti-dementia treatment, respectively.

**Table 1:** Descriptive statistics and correlations matrix.

Variable	Mean	SD	1	2	3	4	5	6	7	8	9	10	11	12	13
1. Age (2010)	67.00	1.68	-												
2. Education (2010)	NA	NA	-.07^*^	-											
3. Income (2010)	37.22	20.53	-.06^*^	.28^***^	-										
4. Health (2010)	4.07	.78	.01	.09^**^	.13^***^	-									
5. Illness (2010)	.98	.13	-.02	.04	-.01	.02	-								
6. Activity (2010)	3.43	.74	.03	.09^**^	.01	.19^***^	.02	-							
7. Volunteering (2010)	2.00	4.33	.09^**^	.07^*^	.08^*^	.09^**^	-.03	-.03	-						
8. COPSOQ (2010)	1.80	.71	-.01	-.01	-.09^**^	-.26^***^	.01	-.01	-.04	-					
9. COPSOQ (2012)	1.81	.68	.01	-.05	-.07^*^	-.21^***^	.01	.02	-.01	.55^***^	-				
10. COPSOQ (2014)	1.81	.68	-.03	-.03	-.09^**^	-.23^***^	-.01	-.06^*^	.03	.54^***^	.61^***^	-			
11. Prescription (2010)	.02	.07	.05	.05	.04	-.04	-.08^**^	.04	.05	.06^*^	.01	.47^***^	-		
12. Prescription (2012)	.02	.09	.06^*^	.01	.02	-.04	-.12^***^	.03	.02	.03	.01	.05	.59^***^	-	
13. Prescription (2014)	.02	.09	.02	.03	.01	-.04	-.12^**^	.03	.03	-.02	.01	.07^*^	.47^***^	.83^***^	-

Notes

N = 1,001

^*^ p < .05

^**^ p < .01

^***^ p ≤ .001

Health refers to self-rated health; Illness refers to previous illness or complaints; COPSOQ refers to self-reported cognitive complaints; Prescription refers to the likelihood of being prescribed an anti-dementia treatment.

### Inferential statistics

#### The estimated normal causation path model

Table 2 reports the standardized path coefficients of the amount of hours spent volunteering in 2010 (discontinuous and continuous) on one’s cognitive complaints and the likelihood of being prescribed an anti-dementia treatment in 2012 and 2014. Results reflect controlling for the above-mentioned background variables and baseline self-reported cognitive complaints and prescribed anti-dementia medication (i.e., previous levels of these outcome variables).

**Table 2:** Standardized coefficients of the normal causation path model: Paths from hours of voluntary work (2010) to cognitive complaints and anti-dementia treatment (2012 & 2014).

Path: Independent variable ➔ Dependent variable	Estimate (S.E.)	*p*
**Discontinuous volunteering group**		
Hours spent volunteering (2010) ➔ Cognitive complaints (2012)	-.019 (.011)	.069
Hours spent volunteering (2010) ➔ Cognitive complaints (2014)	-.033 (.018)	.074
Hours spent volunteering (2010) ➔ Anti-dementia treatment (2012)	-.040 (.036)	.267
Hours spent volunteering (2010) ➔ Anti-dementia treatment (2014)	-.009 (.010)	.347
**Continuous volunteering group**		
Hours spent volunteering (2010) ➔ Cognitive complaints (2012)	-.238 (.026)	< .001
Hours spent volunteering (2010) ➔ Cognitive complaints (2014)	-.080 (.005)	< .001
Hours spent volunteering (2010) ➔ Anti-dementia treatment (2012)	-.057 (.014)	< .001
Hours spent volunteering (2010) ➔ Anti-dementia treatment (2014)	-.063 (.015)	< .001

Notes: *N =* 1,001; S.E. equals standard error.

Our results indicate that volunteering discontinuously versus continuously volunteering was unrelated to changes in self-reported cognitive complaints in 2012 or in 2014. In contrast, retired seniors who volunteered continuously, versus those who did not volunteer or volunteered discontinuously, reported a significant decrease in cognitive complaints in 2012 (Adjusted R^2^ = .26) and 2014 (Adjusted R^2^ = .47). However, the effect of volunteering more hours per week weakened over time as the standardized path coefficient became smaller as the time lag became larger. These results support Hypothesis 1. Most importantly, we found that continuous volunteering, versus not volunteering or discontinuously volunteering, was associated with a 2.44 (95%CI [1.86; 3.21]), and 2.46 (95%CI [1,89; 3.24]) times lower likelihood of being prescribed an anti-dementia treatment in 2012 (Herzberg’s R^2^ = .34) and 2014 (Herzberg’s R^2^ = .29), respectively. These results support Hypothesis 2.

### The estimated reversed causation path model

Table 3 reports the standardized path coefficients of one’s cognitive complaints and the likelihood of being prescribed an anti-dementia treatment in 2010 and 2012 on one’s average amount of hours spent volunteering per week in 2012 and 2014. As with the normal causation path model, we controlled for the above-mentioned background variables and previous levels of the outcome variable. Hence, we estimated change in the outcome variables.

**Table 3:** Standardized coefficients of the reverse causation path model: Paths from cognitive complaints and anti-dementia treatment (2010 & 2012) to hours of voluntary work (2012 & 2014).

Path: Independent variable ➔ Dependent variable	Estimate (S.E.)	*p*
**Discontinuous volunteering group**		
Cognitive complaints (2010) ➔ Hours spent volunteering (2012)	-.019 (.036)	.609
Cognitive complaints (2012) ➔ Hours spent volunteering (2014)	-.048 (.038)	.156
Anti-dementia treatment (2010) ➔ Hours spent volunteering (2012)	-.001 (.039)	.926
Anti-dementia treatment (2012) ➔ Hours spent volunteering (2014)	-.028 (.074)	.702

Notes: *N =* 1,001; S.E. equals standard error.

Our results indicate that neither the level of self-reported cognitive complaints, nor anti-dementia treatments (i.e., likelihood) in 2010 or 2012 were associated with changes in one’s voluntary activities (continuous, discontinuous, or no volunteering) in 2012 or 2014 respectively. Therefore, we conclude that reverse causation is not likely. This is further supported by comparing the BIC values (i.e., full model fit information is not available for models with zero-inflated Poisson variables in Mplus and can thus not be used to determine model fit) of the normal causation path model (BIC = 14500.21) and the reverse causation path model (BIC = 14728.53).

## Discussion

Recently, Anderson and colleagues [17] proposed that the increased social, physical, and cognitive activity that accompanies the enactment of voluntary work might bolster retired seniors from the risk of memory impairment and dementia in later life. However, to date it is unclear whether voluntary work in later life serves as a protective factor for cognitive health. This study focused on self-reported measures of cognitive complaints and on the likelihood of being prescribed an anti-dementia treatments from the Swedish National Prescribed Drug Register. Our results indicated that retired seniors who continuously volunteered reported a significant decrease in their cognitive complaints in 2012 and 2014. In addition, they were less likely to be prescribed an anti-dementia treatment in 2012 and 2014 compared to those who volunteered episodically or those who never volunteered. In contrast, we found that retired seniors who volunteered episodically reported no significant changes in their cognitive complaints over time. In addition, there was no significant relationship with the likelihood of being prescribed an anti-dementia treatment.

In sum, these results support the notion that retired seniors who continuously engaged in voluntary work, compared to those who did not engage or who engaged only episodically in voluntary work are at lower risk for self-reported cognitive complaints and being prescribed an anti-dementia treatment.

### Limitations

Despite its merits, this study it is not without limitations. First, most respondents did not report severe cognitive complaints and relatively few respondents were prescribed an anti-dementia treatment. Although the prevalence rates in our study were slightly smaller than the prevalence rates reported by a recent revision of prevalence rates of dementia in older people in Europe [28], we would like to acknowledge that other studies, conducted in a single European country, found that proportions of pharmacotherapy of dementia was on average 24.6% in seniors aged 65 and above [35]. However, other studies [36], that reviewed the EURODEM publications (dementia studies conducted in the European Union countries) found that crude prevalence rates for dementia varied between 5.9% and 9.4% in subjects aged over 65. These higher prevalence rates for dementia can be partially explained by the fact that our sample consisted of relatively “young” retired participants (mean ages ranged from 67.75 in 2010 to 71.93 in 2014) with a narrow standard deviation; an explanation offered by Bohlken and colleagues [35]. Because the likelihood of being diagnosed with dementia increases substantially with age, it is not surprising that the prevalence of dementia was lower in our sample compared to the study by Bohlken and colleagues [35]. It should be noted that we did account for these lower prevalence rates of dementia in our analyses, which is often not the case in most research on dementia. By doing so, we prevented the lower prevalence rates of dementia from being outliers that may pull the association between anti-dementia treatment (and cognitive complaints) and hours of volunteering. In addition, we were able to study the association between hours of voluntary work and the occurrence of a rare, yet very important, observation such as the likelihood of being diagnosed with dementia. As a consequence, our results are most likely to be at the lower bound of the true association between continuous engagement in voluntary work and reduced cognitive complaints and anti-dementia treatments.

Second, as with all longitudinal research, there is always the possibility of reverse causation. However, the results from our reverse causation path model indicated that this is highly unlikely. Moreover, and related to this limitation, there is always the possibility that those with major neurocognitive impairment and/or severe dementia are not able to fill out the questionnaire. Respondents who were prescribed an anti-dementia treatment in our sample were indeed those individuals who “only” suffered from mild to moderate dementia (low to moderate strength of the daily dosage of the prescribed anti-dementia treatments) and reported moderate cognitive complaints (ranging from 2.15 to 2.44 on a 4-point scale). Future research could try to overcome this potential selection bias by having primary care physicians or family caregivers complete a report about the patient’s cognitive complaints and problems. Previous studies have shown that this method, in combination with a patient test (physician diagnosis of dementia), results in an accurate prediction of which patients suffer from a true degenerative disease that may cause dementia [37].

Third, although having registry indicators of dementia treatments is favorable, it is noteworthy that these data are only available for those individuals who, once prescribed an anti-dementia treatment, actually redeemed this prescription. Hovstadius and Petersson [38] estimated that approximately 3% of all Swedes fail to redeem their prescription.

Finally, we were unable to omit a ‘frame shift’ option in the comparisons respondents used when assessing their level of cognitive complaints. Specifically, this implies that respondents might think about their level of cognitive complaints relative to the level of cognitive complaints of those in their surroundings who are not receiving an anti-dementia treatment. Consequently, there might be problems with floor and ceiling effects in the measurements of this concept [39]. Therefore, we suggest future research to modify the measure of self-reported cognitive complaints in such a way that it measures self-assessed change between two measurement moments.

### Avenues for future research

Future research could try to unravel the constellation of voluntary work characteristics that are most likely to be associated with few cognitive complaints and lower dementia prevalence among senior volunteers. Depending on the nature of the voluntary task, one will have more social contact, require higher levels of physical mobility, or tap into varying degrees of cognitive skills. These varying levels of social contact, and physical and cognitive activity might explain why certain types of voluntary work are more likely to be associated with few cognitive complaints and low dementia prevalence. In addition, we call for brain-imaging and physiological studies to investigate potential mediating mechanisms in the proposed relationship between volunteering in later life and cognitive health. We suggest that the mental stimulation provided by voluntary work could increase synaptogenesis in adulthood and add new neurons, whereas the physical activity associated with voluntary work could enhance non-neural components of the brain [40, 41]. Both mechanisms were found to impact on the development of dementia pathology in late-adults [38, 40]. In addition, voluntary work has been related to reduced risks of cardiovascular diseases and hypertension [42, 43]. Because Qiu and colleagues [44] have indicated that vascular disorders, such as cardiovascular disease and hypertension, are involved in the pathogenesis and progression of dementia in later life, these associations might explain why continuously volunteering was associated with lower self-reported cognitive problems and a decreased likelihood to be prescribed an anti-dementia treatment.

### Practical implications

Our study underlined the pivotal role of voluntary work in later life in terms of self-reported cognitive problems and the likelihood of being prescribed an anti-dementia treatment. Based on our results it can be concluded that motivating retired seniors to engage in voluntary work on a continuous basis would help to improve their cognitive health. Therefore, we suggest several ways organizations and society could motivate retired seniors to volunteer, what factors they should take into account when promoting voluntary work, and how society could help organizations to attract senior volunteers.

First, creating a culture of volunteering is at the heart of any effort to boost voluntary work among retired seniors and should therefore form the backbone of any volunteer recruitment campaign [45]. To entrench volunteering as a key driver toward a better cognitive health, we would like to note the need for a life course approach to volunteering; promoting and encouraging volunteerism throughout the lifespan. Hence, we point toward programs promoting voluntary work as part of the school’s curriculum, as well as toward programs that connect voluntary work among different generations [46]. In these programs, retired seniors could share their expertise (1) by becoming a board member, (2) as a mentor or coach for younger generations, or (3) by mobilizing community action. These suggestions provide retired seniors with the opportunity to share wisdom and experience with younger generations, while simultaneously allowing them to learn new skills from younger generations. Hence, we suggest developing and implementing information campaigns to promote voluntary work among retired seniors.

Second, organizations should account for other factors when attracting and retaining senior than when attracting and retaining younger volunteers. For example, with aging comes the issue of transportation and mobility. Transportation to and from the voluntary work can become a significant barrier to retired seniors due to (travel) costs, lack of access to public transportation, and reduced physical mobility [47, 48]. Organizations could, for example, reimburse transportation costs or provide free transportation. In addition, we suggest to make volunteer placements more accessible to all and to be adaptive so as to meet the changing physical abilities of senior volunteers.

Finally, we would like to point out that not only senior volunteers require extra support, but so do the organizations that attract senior volunteers. This includes improvement of the recruitment and management of senior volunteers [49]. For example, it could involve grants to improve and disseminate existing volunteer recruitment and management tools, grants to improve an organization’s willingness to recruit senior volunteers, and funding for campaigns to increase awareness among retired seniors that volunteering in later life is beneficial for one’s cognitive health. In addition, organizations should be made aware that these senior volunteers can serve as mentors for the younger volunteers. Recruiting and retaining these senior volunteers requires volunteer coordinators who are trained to assist in adapting voluntary work to the needs and the physical and cognitive abilities of the individual senior volunteers [49]. However, to date, most non-profit organization have limited financial or human resources to either hire a volunteer coordinator or to provide this coordinator with the required training and education. Therefore, we call for additional efforts and funds to recruit and train volunteer coordinators.
